# An implementation analysis of a quality improvement project to reduce cesarean section in Brazilian private hospitals

**DOI:** 10.1186/s12978-024-01773-6

**Published:** 2024-04-26

**Authors:** Jacqueline Alves Torres, Tatiana Henriques Leite, Thaís Cristina Oliveira Fonseca, Rosa Maria Soares Madeira Domingues, Ana Claudia Figueiró, Ana Paula Esteves Pereira, Mariza Miranda Theme-Filha, Bárbara Vasques da Silva Ayres, Oliver Scott, Rita de Cássia Sanchez, Paulo Borem, Maria Carolina de Maio Osti, Marcos Wengrover Rosa, Amanda S. Andrade, Fernando Maia Peixoto Filho, Marcos Nakamura-Pereira, Maria do Carmo Leal

**Affiliations:** 1 Institute for Healthcare Improvement, Brasília, Brazil; 2 State University of Rio de Janeiro, Social Medicine Institute, Rio de Janeiro, Brazil; 3 Department of Statistical Methods, Federal University of Rio de Janeiro, Rio de Janeiro, Brazil; 4 Oswaldo Cruz Foundation . National Institute of Infectious Disease Evandro Chagas, Rio de Janeiro, Brazil; 5 Oswaldo Cruz Foundation, Rio de Janeiro, Brazil; 6 Cambridge Imperial, London, UK; 7 Hospital Israelita Albert Einstein, São Paulo, Brazil; 8 Hospital da Luz, São Paulo, Brazil; 9 Hospital Moinhos De Vento, São Paulo, Brazil; 10 Pasteur Hospital, Rio de Janeiro, Brazil; 11 Oswaldo Cruz Foundation, National Institute of Health for Women, Children and Adolescents Fernandes Figueira, Rio de Janeiro, Brazil

**Keywords:** Vaginal birth, Implementation analysis, Bayesian analysis

## Abstract

**Background:**

Brazil has one of the highest prevalence of cesarean sections in the world. The private health system is responsible for carrying out most of these surgical procedures. A quality improvement project called Adequate Childbirth Project (“Projeto Parto Adequado”- PPA) was developed to identify models of care for labor and childbirth, which place value on vaginal birth and reduce the frequency of cesarean sections without a clinical indication. This research aims to evaluate the implementation of PPA in private hospitals in Brazil.

**Method:**

Evaluative hospital-based survey, carried out in 2017, in 12 private hospitals, including 4,322 women. We used a Bayesian network strategy to develop a theoretical model for implementation analysis. We estimated and compared the degree of implementation of two major driving components of PPA—“Participation of women” and “Reorganization of care” – among the 12 hospitals and according to type of hospital (belonging to a health insurance company or not). To assess whether the degree of implementation was correlated with the rate of vaginal birth data we used the Bayesian Network and compared the difference between the group “Exposed to the PPA model of care” and the group “Standard of care model”.

**Results:**

PPA had a low degree of implementation in both components “Reorganization of Care” (0.17 – 0.32) and “Participation of Women” (0.21 – 0.34). The combined implementation score was 0.39–0.64 and was higher in hospitals that belonged to a health insurance company. The vaginal birth rate was higher in hospitals with a higher degree of implementation of PPA.

**Conclusion:**

The degree of implementation of PPA was low, which reflects the difficulties in changing childbirth care practices. Nevertheless, PPA increased vaginal birth rates in private hospitals with higher implementation scores. PPA is an ongoing quality improvement project and these results demonstrate the need for changes in the involvement of women and the care offered by the provider.

## Introduction

Cesarean section (CS) can save the lives of women and newborns and there is evidence of a higher risk of maternal and neonatal mortality in countries with a prevalence of cesarean section below 10% [1]. However, there is no evidence of benefits from cesarean rates greater than 15% at the population level [2] and there are many negative short and long-term consequences of performing a cesarean section without clinical indication, for both mothers [3–5] and babies [6–9] even when adjustments are made for maternal and/or fetal risk. Moreover, evidence suggests that cesarean section could be associated with prematurity [10, 11] and early term births [10, 12].

Brazil has one of the highest prevalence of cesarean section (CS) in the world. Currently, according to data from the Brazilian Information System on Live Births (SINASC), 57,2% of births in 2020 were carried out via CS [13]. Based on the C-model tool [14], and considering demographic and obstetric characteristics of Brazilian women, the expected prevalence of cesarean sections should not exceed 25% of total births. Nevertheless, the actual rate is over twice this value. In addition to the high prevalence of cesarean sections, there is considerable disparity between the public and private healthcare sectors in Brazil, with private hospitals presenting twice as many CS. Hence, strategies for reducing CS without clinical indication should be primarily focused on the private sector.

In 2014, the National Agency for Supplementary Health (acronyms in Portuguese—ANS), a state body responsible for regulating the Brazilian health insurance market, developed a quality improvement project [15] called “Projeto Parto Adequado”- PPA (Adequate Childbirth Project), in partnership with the Institute for Healthcare Improvement (IHI), the Israelita Albert Einstein Hospital (HIAE), and with the support of the Brazilian Ministry of Health.

The PPA is a complex and multifactorial quality improvement initiative whose main objective is to identify innovative and viable models of care for labor and childbirth that promote vaginal birth and reduce the frequency of cesarean sections without clinical indication in the private health system [16]. The PPA was implemented in three phases: i) phase 1 (2015 – 2016) tested the intervention and involved 35 (12 public and 23 private) hospitals, including 19 health plan operators; ii) phase 2, started in May 2017 and ongoing, extended the project to a variety of providers and health operators; and iii) phase 3, launched in October 2019 and still ongoing, aimed at promoting effective strategies to improve the quality of childbirth care on a large scale, with the possibility of including the set of maternity hospitals and operators in Brazil [17].

The PPA targeted improvements across four components: 1) governance: forming a coalition between leadership in the health sector, aligning quality and safety in labor and childbirth care; 2) participation of women and families: empowering women and families so they actively participate in the entire process of pregnancy, birth, and postpartum care; 3) reorganization of care: reorganizing the model of childbirth care to favor the physiological evolution of labor and ensuring that CS is based on clinical criteria; 4) monitoring: structuring information systems that allow lifelong learning [16].

Briefly, the PPA uses the IHI improvement model, where through the cyclical and incremental implementation of changes, the proposed activities are tested and adjusted to the local context [18]. In the first phase of the project, managers and local leaders participated in face-to-face and virtual learning sessions, which aimed to train the improvement model, carry out the initial tests of change to reduce the caesarean rate based on the four PPA components and share successful experiences and challenges in implementing changes. In addition, the project offered clinical training at realistic simulation centers, with a focus on assisting physiological vaginal delivery and managing obstetric complications [19]. At the hospital level, the project implemented new forms of care organization, which included changes in the hospital environment, participation of nurse-midwives in childbirth care and implementation of clinical guidelines. Activities for women included access to information, participation in educational groups, encouragement to develop a birth plan and visit to the hospital. More information about the PPA is available at the ANS website [17] and at Boren et al. [19].

This article has three main objectives: 1) to evaluate the degree of implementation of the components “Reorganization of care” and “Participation of women”, separately and combined; 2) to evaluate the association between the type of hospital (owned by a health insurance company or not) and the implementation of these two components; 3) to assess whether the degree of implementation of these two components was associated with vaginal birth rates. Our hypothesis is that the degree of implementation of the components “Reorganization of care” and “Participation of women” are different, that the degree of implementation varies according to the hospital type and that the degree of implementation affects the rate of vaginal birth.

## Methods

### Study design

Cross-sectional hospital-based evaluative study (the “Healthy Birth” study), using quantitative data collected from March 2017 to August 2017, 6 to 8 months after the end of the first phase of PPA.

### Sample design

Convenient sample of twelve private hospitals among the 23 included in the first phase of PPA. The convenient sample was based on three contextual criteria that could have affected the implementation of the planned activities: the hospital location (according to Brazilian regions); the type of hospital (hospitals owned or not owned by health insurance companies); and hospital performance (hospital performance was classified as “good” or “poor”, according to the evaluation of the PPA coordination team [16]).

Only hospitals in the Northeast, Southeast and South regions were included, as hospitals located in the North and Midwest regions did not participate in the first phase of PPA. The Brazilian private healthcare system is composed of two types of hospitals: hospitals that belong to a health insurance company and hospitals that do not belong to a health insurance company. Both provide assistance to users of health plans and to users who pay for services by direct disbursement.

In each of the 12 hospitals, 400 women were included. This planned sample size aimed to detect a 10% reduction in the proportion of CS, considering an estimate of 50%, 80% power, and 5% significance levels.

### Study population

All women admitted to the selected maternity hospitals who had a live birth (of any gestational age or birth weight) or a stillbirth (with gestational age ≥ 22 weeks and/or birth weight ≥ 500 g), were eligible for the study.

Exclusion criteria included women who gave birth before admission to the hospital; women with extreme communicating difficulty (such as foreigners who could not understand Portuguese, women with hearing and speaking impairments, and women with mental or neurological diseases suffering severe cognitive impairment); and women who legally terminated their pregnancy.

### Data collection

We carried out face-to-face interviews with eligible women in the post-partum period, at least six hours after vaginal birth and twelve hours after CS. All eligible women were consecutively invited to participate, until we enrolled 400 participants in each hospital. In total, we interviewed 4,798 women. The interview included questions on maternal identification; socio-economic condition; previous obstetric history; maternal anthropometric data; prenatal care; illnesses and medication during gestation, labor, and birth; and evaluation of childbirth care received by the woman and newborn. We also extracted data from medical records of women and neonates after hospital discharge. All questionnaire used was published in Torres, 2018 [16].

We used electronic forms (REDCap) in all interviews. Women signed an informed written consent before the interview.

## Theoretical model

**Fig. 1:**
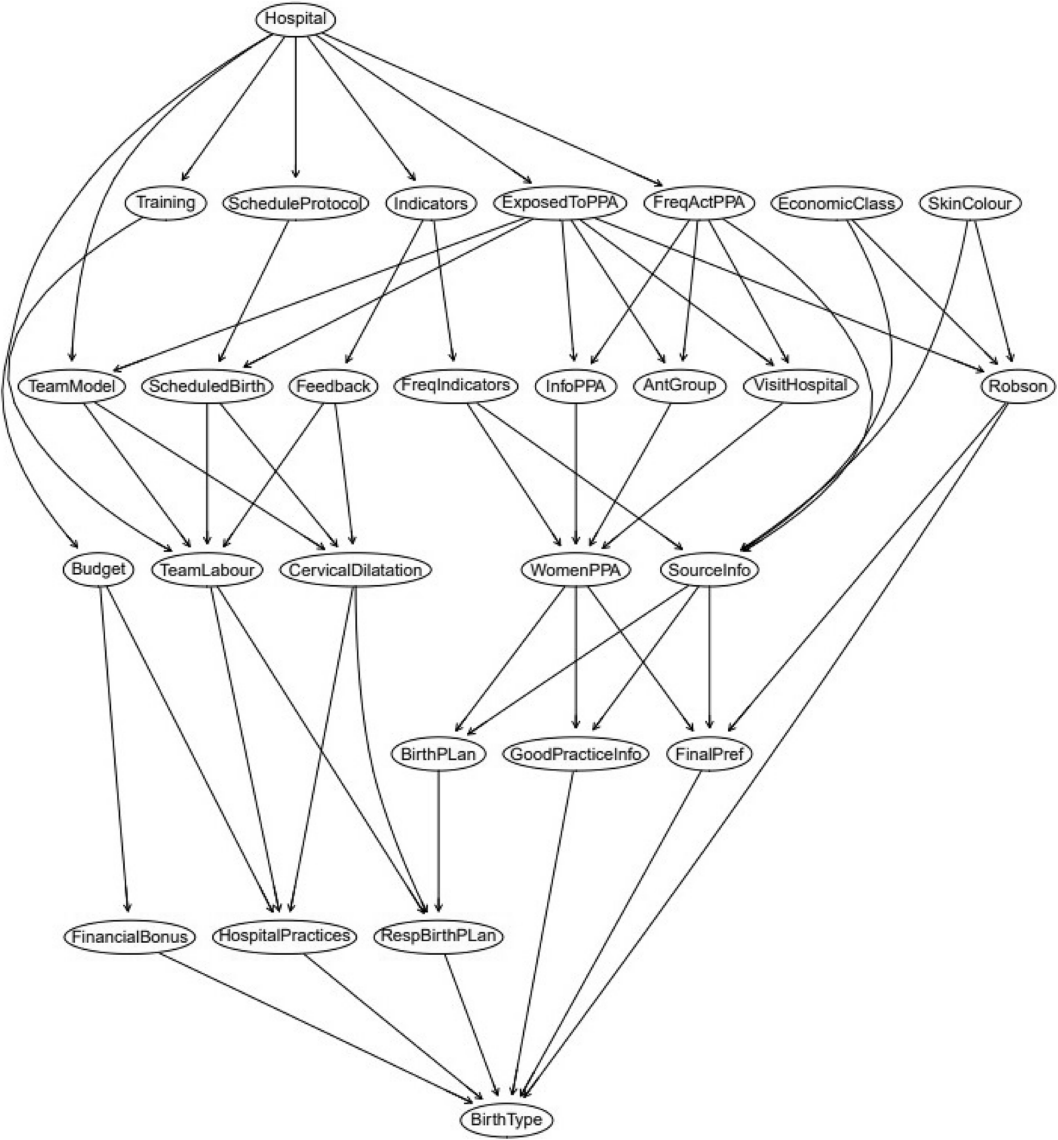
Birth network used for the implementation analysis. Please see Table 1 for definition of variables

To assess the implementation of PPA, we used “The Birth Network” (Fig. 1) — a theoretical model developed by the research team after consulting experts on the topic including obstetricians, nurses/midwives, and epidemiologists. We opted for a theoretical model, rather than a data-driven one, because the PPA is based on scientific evidence [20] and on 2 successful strategies for reducing caesarean sections in Brazilian private hospitals [21, 22]. Therefore, the network considered the four driving components of PPA (Governance, Participation of women, Reorganization of care, Monitoring) [16] and potential confounders of the effect of PPA in reducing cesarean sections. All the variables used in the birth network are described in Table 1.

**Table 1:** Birth network variables

Variable name	**Variable description**	**Answer categories**	**Source of data**
I – Governance			
Training	Whether the hospital staff participated in training offered by the Open School Institute for Healthcare Improvement, Sofia Feldman Hospital, and Albert Einstein Hospital	“All”—for three instances of training; “Two or less”—for 0 to 2 instances of training	Management interview
Financial Bonus	Whether the hospital uses a financial bonus strategy to implement protocols and routines	Yes, No	Management interview
Budget	Whether the hospital has a budget to improve maternal and childcare	Yes, No	Management interview
II) Participation of women			
Good Practice Info	Whether women received information during pregnancy about: 1)signs of labor, 2)signs of risk danger, and 3) best practices during labor	None – received no information; Yes – received at least one information	Interview with women
Final Pref	Women’s final preference of the type of birth	Vaginal; Cesarean/no preference	Interview with women
Ant Group	Whether women were offered to participate in an antenatal group activity	Yes, No	Interview with women
Visit Hospital	Whether women were offered to visit the hospital where they gave birth	Yes, No	Interview with women
Info PPA	Whether women knew that the hospital was a PPA participant	Yes, No	Interview with women
Women PPA	Variable composed of three items: 1)if the participation of the hospital in the PPA was important for the woman’s choice of this hospital for birth; 2) if the woman visited the hospital before birth; 3) if the woman participated in a hospital antenatal group	All – to women who responded “yes” to the three questions; and two or less – to women who responded “yes” to two or fewer questions	Interview with women
Source Info	Whether information about best practices was provided by the hospital/insurance company, or from other sources	Hospital/Health Insurance Company; Other sources	Interview with women
Freq Act PPA	Frequency of publication of PPA activities to women/clients	Regular or Non-regular	Management interview
Birth Plan	Whether the woman prepared a birth plan	Yes, No	Interview with women
III) Reorganization of care:			
Team Model	Type of healthcare team who provided labor and childbirth care	Hospital Staff, External and Hospital Staff, only External Staff	Interview with women
Team Labor	Type of healthcare who provided labor and childbirth care	Doctor, Nurse and Doctor, No Labor	Interview with women
Schedule Protocol	Existence of a protocol for scheduling cesarean sections according to gestational age at birth	Yes, No	Management interview
Scheduled Birth	Whether the woman had a scheduled birth	Yes, No	Interview with women
Cervical Dilatation	Cervical dilation upon hospital admission	No Labor, < 4 cm, > = 4 cm	Medical records
Resp Birth Plan	Whether the woman’s birth plan was respected	Respected, Not Respected, No Birth Plan	Interview with women
Hospital Practices	Whether the woman had access to best practices during labor (oral fluids, freedom of movement, shower, non-pharmacological methods of pain relief)	< 4 items, > = 4 items, No Labor	Interview with women
IV) Monitoring			
Indicators	Whether the hospital monitors the following indicators: cesarean rate, cesarean rate by Robson group, childbirth care by nurses/midwives, vaginal birth with episiotomy, admission to Neonatal Intensive Care Unit, proportion of early-term births (37–38 gestational weeks)	< = 4 items, 5 or 6 items	Management interview
Feedback	Identifying which professionals gathered feedback on results of perinatal indicators	Each doctor individually; Doctors and Team; Doctors, Team and User	Management interview
Freq Indicators	Frequency of feedback regarding perinatal indicators	No frequency, Regular, irregular, hospital does not monitor indicators	Management interview
V) Confounders			
Economic Class^32^	Brazilian economic classification	A1/A2, B1/B2, and C1/C2 (where “A” represents the highest economic class)	Interview with women
Skin Colour	Self-reported skin colour of women	White, Non-White	Interview with women
Robson^14^	Classification of women into Robson groups 1 to 4	Yes, No	Medical records
VI) Exposed			
Exposed To PPA	Whether woman was targeted by the PPA model of care	Yes, No	Management Interview
VII) Context			
Hospital	Whether the hospital belonged to a health insurance company	Yes, No	Management interview
VIII) Outcome			
Birth Type	Type of birth	Vaginal/forceps, Cesarean section	Interview with women

This paper analyzes the implementation of the components “Reorganization of care” and “Participation of women”. The components “Monitoring” and “Governance” were assessed through an interview with hospital managers (data not presented) and the small number of observations and the variability of data prevented an isolated implementation analysis of these components. However, all network data was used to estimate the predicted probability of vaginal birth.

The analysis included 4,322 women with complete dataset and was carried out according to the classification of women as “Exposed to the PPA model of care” and as exposed to the “Standard of care model”. Women “Exposed to the PPA model of care” varied in each hospital. In two hospitals, the target population of PPA was composed of all primiparous women; in two others, by women in Robson groups 1 to 4; and in 8 hospitals, by women admitted by the hospital's on-call staff. Women in the “PPA model of care” would be exposed to the activities advocated by the quality improvement project, which includes: access to information during pregnancy; visits to the maternity hospital; preparation of the birth plan by the pregnant woman; encouragement of labor; labor and childbirth care as per the collaborative doctor/nurse-midwife model; and use of best practices [16]. Women in the “Standard of care model” were assisted according to the current practice in Brazilian private hospitals, characterized by the same doctor being responsible for prenatal and childbirth care; low participation of nurses/midwives; high proportion of antepartum cesarean section; and high levels of intervention in labor and childbirth care [23].

**Fig. 2:**
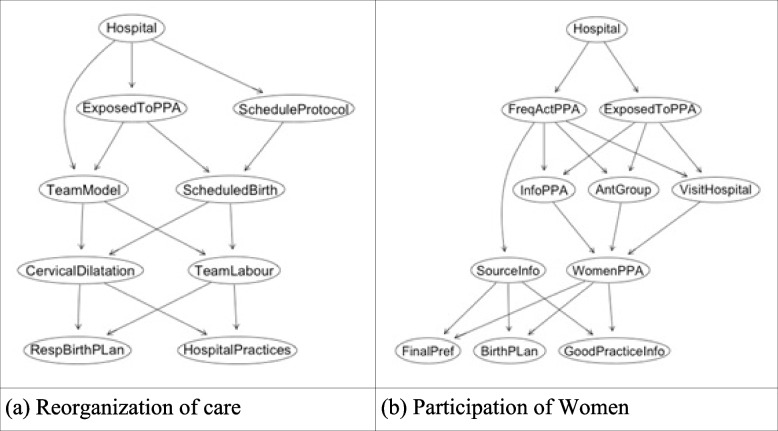
Subgraphs of birth network used for implementation analysis of each component. Please see Table 1 for definition of variables

Figure 2 represents the two components—“Reorganization of care” and “Women participation”- in isolation. For “Reorganization of care”, implementation is measured by analyzing the differences between the two models of care according to the outcomes “RespBirthPlan” (Respected birth plan) and “HospitalPractices” (access to oral fluids, freedom of movement, shower, and non-pharmacologic methods of pain relief) (Fig. 2A). For “Participation of women” (Fig. 2B), the main outcomes are “FinalPref" (woman´s final preference of the type of birth), "GoodPracticeInfo" (information received during pregnancy about signs of labor, signs of risk danger, and best practices during labor) and "BirthPlan" (preparation of a birth plan during pregnancy).

We used a Bayesian Network (BN) analysis. We defined topology using expert knowledge. The conditional probabilities defining the BN were estimated using the Bayesian paradigm for inference and prediction. The sampling model assumed for the local distributions was multinomial, as all the variables composing the network were discrete. This structure will allow for causal inference of the variables in the network. Predictive probabilities were obtained by logic sampling [24]. The query will represent a fixed value for the variables in the driving component under evaluation. The consequences of intervention *E* on query *Q* allowed us to measure the impact of changes in the system. Given a graph *G* and set of evidence *E*, the probability of specific queries can be obtained by setting the parameters in the MAP estimator. For details on BN concepts and estimation, see Heckerman et al. [25].

$$P\left({Y}_{Q}=y \left|E, G\right.\right)$$

To address the first objective, we assessed the degree of implementation of “Reorganization of care” and “Participation of women” separately and in combination. We then compared the results across 12 hospitals. We created a "Model-Hospital'' to represent all women with the best level of each variable composing the subgraphs in question. Analogously, we created the "Null-Hospital'', which represented all women with the worst categories of each variable composing the subgraph. The scores were computed for these hypothetical hospitals such that a scale was available for comparing the performance of each hospital.

To investigate whether the type of hospital (owned by a health insurance company or not) could influence the implementation of PPA (objective 2), we computed the score for the “Reorganization of care” and “Participation of women” according to the type of hospital. Finally, to determine whether the degree of implementation was correlated with the rate of vaginal birth (objective 3), we estimated the probabilities of vaginal birth among all women by using the network and considering the difference between groups “Exposed to the PPA model of care” compared to the “Standard care model”. To test for covariation we included a measure of linear correlation between the components “Participation of women” and “Reorganization of care” and between the difference in the probability of vaginal birth and the total score of implementation of both components.

## Results

Table 2 shows the predicted probability of each variable used in the Birth network model. Women “Exposed to the PPA model of care” had a higher probability of all indicators pertaining to the “Participation of women” and “Reorganization of care” components, when compared to the “Standard of care model”. No difference was observed in confounding variables between these groups. A unique exception was the Robson classification. Groups 1 to 4 appeared with greater frequency among women “Exposed to the PPA model of care”.

**Table 2:** Predicted probabilities of all variables in the birth network

Indicator	Description	Total	Exposed to PPA	Standard of Care Group	Significance level^1^
**Governance**					
**Training**					
All	Whether the hospital staff participated in training offered by the Open School Institute for Healthcare Improvement, Sofia Feldman Hospital, and Albert Einstein Hospital	2	-	-	-
Partial		10			
**Financial Bonus**					
No	Whether the hospital uses a financial bonus strategy to implement protocols and routines	8	-	-	-
Yes		4			
**Budget**					
No	Whether the hospital has a budget to improve maternal and childcare	4	-	-	-
Yes		8			
**Participation of Women**					
**Good Practices Info**					
None	Whether women received information during pregnancy about: 1)signs of labor, 2)signs of risk danger, and 3) best practices during labor	1113	25,3 (24,6 – 26,2)	25,7 (24,8 – 26,7)	*
At least one		3209	74,7 (73,6 – 75,6)	74,3 (73,3 – 75,2)	
**Final Pref**					
Vaginal	Women’s final preference of the type of birth	2075	54,1 (52,9 – 55,4)	43,7 (42,7 – 44,9)	**
Cesarean/No preference		2247	45,9 (44,5 – 46,8)	56,3 (55,2 – 57,4)	
**Ant Group**					
No	Whether women were offered to participate in an antenatal group activity	2854	64,2 (63,3 – 65,4)	67,6 (66,7 – 68,5)	**
Yes		1468	35,8 (34,8 – 36,9)	32,4 (31,3 – 33,2)	
**Visit Hospital**					
No	Whether women were offered to visit the hospital where they gave birth	2126	47,3 (46,3 – 48,4)	51,0 (50,0 – 52,1)	**
Yes		2196	52,7 (51,7 – 53,6)	49,0 (48,5 – 50,0)	
**Info PPA**					
No	Whether women knew that the hospital was a PPA participant	2753	62,8 (61,7 – 63,6)	64,7 (63,7 – 65,5)	**
Yes		1569	37,2 (36,5 – 38,2)	35,3 (34,2 – 36,2)	
**Women PPA**					
No	Variable composed of three items: 1)if the	2631	59,8 (58,9 – 60,8)	62,6 (61,4 – 63,6)	**
Yes	participation of the hospital in the PPA was important for the woman’s choice of this hospital for birth; 2) if the woman visited the hospital before birth; 3) if the woman participated in a hospital antenatal group	1691	40,2 (38,9 – 41,4)	37,4 (36,5 – 38,5)	
**Source Info**					
Hospital/ Insurance	Whether information about best practices was provided by the hospital/insurance company, or from other sources	277	8,5 (7,8 – 8,9)	7,9 (7,3 – 8,4)	*
Others		4045	91,5 (91,0 – 91,1)	92,1 (91,4 – 92,5)	
**Freq Act PPA**					
Regular	Frequency of publication of PPA activities to women/clients	9	-	-	-
Irregular		3			
**Birth Plan**					
No	Whether the woman prepared a birth plan	3909	73,8 (72,4 – 74,9)	82,8 (82,0 – 83,7)	**
Yes		413	26,2 (25,0 – 27,2)	17,2 (16,4 – 17,9)	
**Reorganization of Care**					
**Team Model**					
Hospital staff	Type of healthcare team who provided labor and childbirth care	1532	62,5 (61,3 – 63,5)	9,9 (9,2 – 10,3)	
External/Hospital staff		355	7,9 (7,1 – 8,3)	8,5 (8,0 – 9,3)	**
External		2435	29,6 (28,5 – 30,5)	81,6 (80,8 – 82,4)	
**Team Labor**					
Doctor	Type of healthcare who provided labor and childbirth care	750	26,5 (25,5 – 27,4)	9,0 (8,4 – 9,6)	**
Doctor/ Nurse		710	20,5 (19,5 – 21,3)	13,6 (12,9 – 14,2)	
No labor		2862	53,0 (52,0 – 54,1)	77,4 (76,3 – 78,2)	
**Schedule Protocol**					
No	Existence of a protocol for scheduling cesarean sections according to gestational age at birth	1			
> 39 weeks		9	-	-	-
> 40 or 41 weeks		2			
**Scheduled Birth**					
No	Whether the woman had a scheduled birth	2445	76,5 (75,6 – 77,4)	37,7 (36,7 – 38,5)	**
Yes		1877	23,5 (22,6 – 24,5)	62,3 (61,3 – 63,4)	
**Cervical Dilatation**					
< 4	Cervical dilation upon hospital admission	288	10,5 (8,5 – 11,8)	7,5 (6,27 – 9,1)	
> = 4		1172	36,4 (35,5 – 38,2)	15,1 (14,1 – 15,9)	**
No labor		2852	53,1 (52,0 – 54,1)	77,4 (76,3 – 78,2)	
**Resp Birth Plan**					
Respected	Whether the woman’s birth plan was respected	361	15,7 (14,9 – 16,5)	10,7 (10,0 – 11,2)	
Not Respected/partially		52	10,6 (9,7 – 11,2)	6,1 (5,8– 7,0)	**
No Birth Plan		3909	73,7 (72,4 – 74,9)	82,8 (82,0 – 83,7)	
**Hospital Practices**					
< 4 recommended	Whether the woman had access to best practices during labor (oral fluids, freedom of movement, shower, non-pharmacological methods of pain relief)	645	24,0 (22,4 – 25,3)	11,3 (10,6 – 12,0)	
> = 4 recommended		815	23,0 (22,1 – 23,9)	11,3 (10,1 – 12,6)	**
No labor		2862	53,0 (52,0 – 54,1)	77,4 (76,3 – 78,2)	
**Monitoring**					
**Indicators**					
< = 4	Whether the hospital monitors the following indicators: cesarean rate, cesarean rate by Robson group, childbirth care by	2	-	-	-
> 4	nurses/midwives, vaginal birth with episiotomy, admission to Neonatal Intensive Care Unit, proportion of early-term births (37–38 gestational weeks)	10			
**Freq Indicators**					
No frequency	Frequency of feedback regarding perinatal indicators	6			
Regular		1	-	-	-
Irregular		3			
Does not monitor indicators		2			
**Feedback**					
Each Doctor	Identifying which professionals gathered feedback on results of perinatal indicators	6	-	-	-
Doctors + Team		3			
Doctors + Team + User		3			
**Confounders**					
**Economic Class**					
A/A2	Brazilian economic classification	944	21,9 (21,1 – 22,9)	21,9 (21,0 – 22,8)	
B1/B2		2454	56,7 (55,2 – 57,9)	56,7 (55,7 – 58,0)	NS
C1/C2		924	21,4 (20,3 – 22,1)	21,4 (20,2 – 22,1)	
**Skin Colour**					
White	Self-reported skin colour of women	2780	63,1 (62,0 – 64,0)	63,2 (62,1 – 64,2)	NS
Non-white		1610	36,9 (35,8 – 37,7)	36,8 (35,9 – 37,8)	
**Robson**					
1–4	Classification of women into Robson groups 1 to 4	2473	79,4 (78,2 – 80,1)	37,7 (36,7 – 38,8)	**
5–10		1849	20,6 (19,8 – 21,9)	62,3 (31,2 – 63,7)	

Legend: ^1^NS= Bayesian Confidence Interval coincide indicating the effects are equal; high significance = the Bayesian Confidence Interval do not intercept indicating the probability of equal effects is small or zero; low significance = the Bayesian Confidence Interval intercept partially indicating the probability of equal effects is moderate

*NS* not-significant

*low significance; ** high significance

**Fig. 3:**
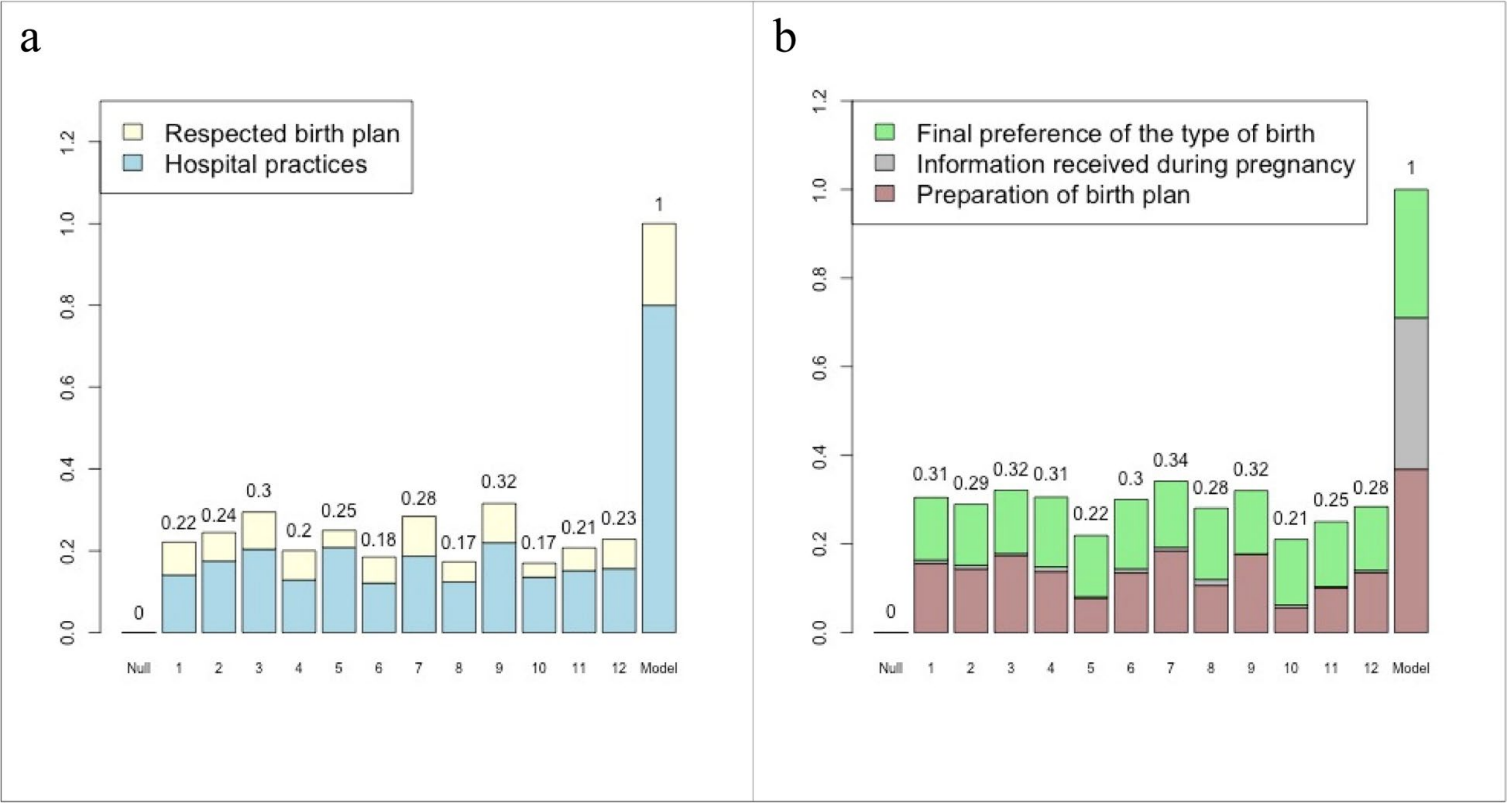
**a** Standardized score of “Reorganization of care”; (**b**) Standardized score of “Participation of women”. Please see Table 1 for more detailed definition of variables. Null = hypothetical hospital where all women had the worst categories of each variable composing the subgraph; Model = hypothetical hospital where all women had the best level of each variable composing the subgraphs in question

All the twelve hospitals achieved low scores according to the “Reorganization of care” (Fig. 3a) and “Participation of women” (Fig. 3b). All scores are standardized so that the Model-Hospital achieves a score of 1 and the Null-Hospital has a score of zero. Hospitals 3, 7, and 9 presented the largest scores (0.30, 0.28, and 0.32) in “Reorganization of care”, while hospitals 6, 8 and 10 presented the worst scores (0.18, 0.17 and 0.17) (Fig. 3a). In “Participation of women”, hospitals 3, 7 and 9 presented the highest scores (0.32, 0.34 and 0.32), while hospitals 5, 10 and 11 presented the lowest scores (0.22, 0.21 and 0.25).

**Fig. 4:**
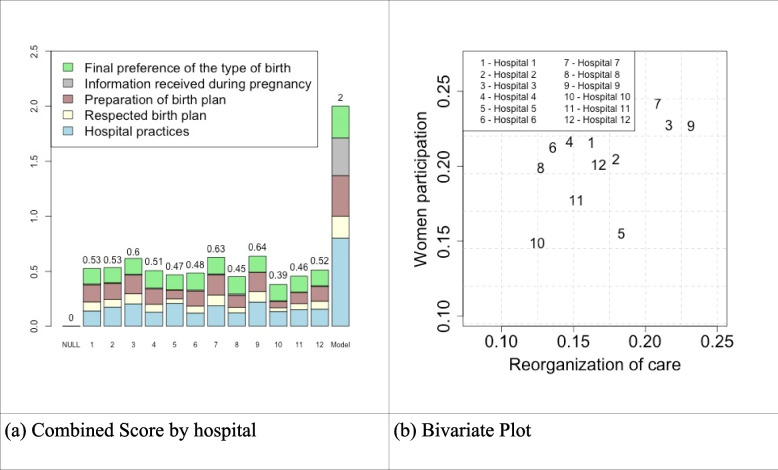
Combined PPA implementation score. Please see Table 1 for more detailed definition of variables. Null = hypothetical hospital where all women had the worst categories of each variable composing the subgraph; Model = hypothetical hospital where all women had the best level of each variable composing the subgraph in question

The combined score of the two components (“Reorganization of care” combined with “Participation of women”) varied from 0.39 (hospital 10) to 0.64 (hospital 9) (Fig. 4a). Hospitals 3, 7, and 9 had the best-combined score (0.60, 0.63, and 0.64), and hospitals 8 and 10 (0.45, 0.39) the worst. In Fig. 4b the score of both components in each hospital is displayed in a bivariate plane. It shows that “Reorganization of care” and “Participation of women” have a positive covariation (correlation of 0.53).

**Fig. 5:**
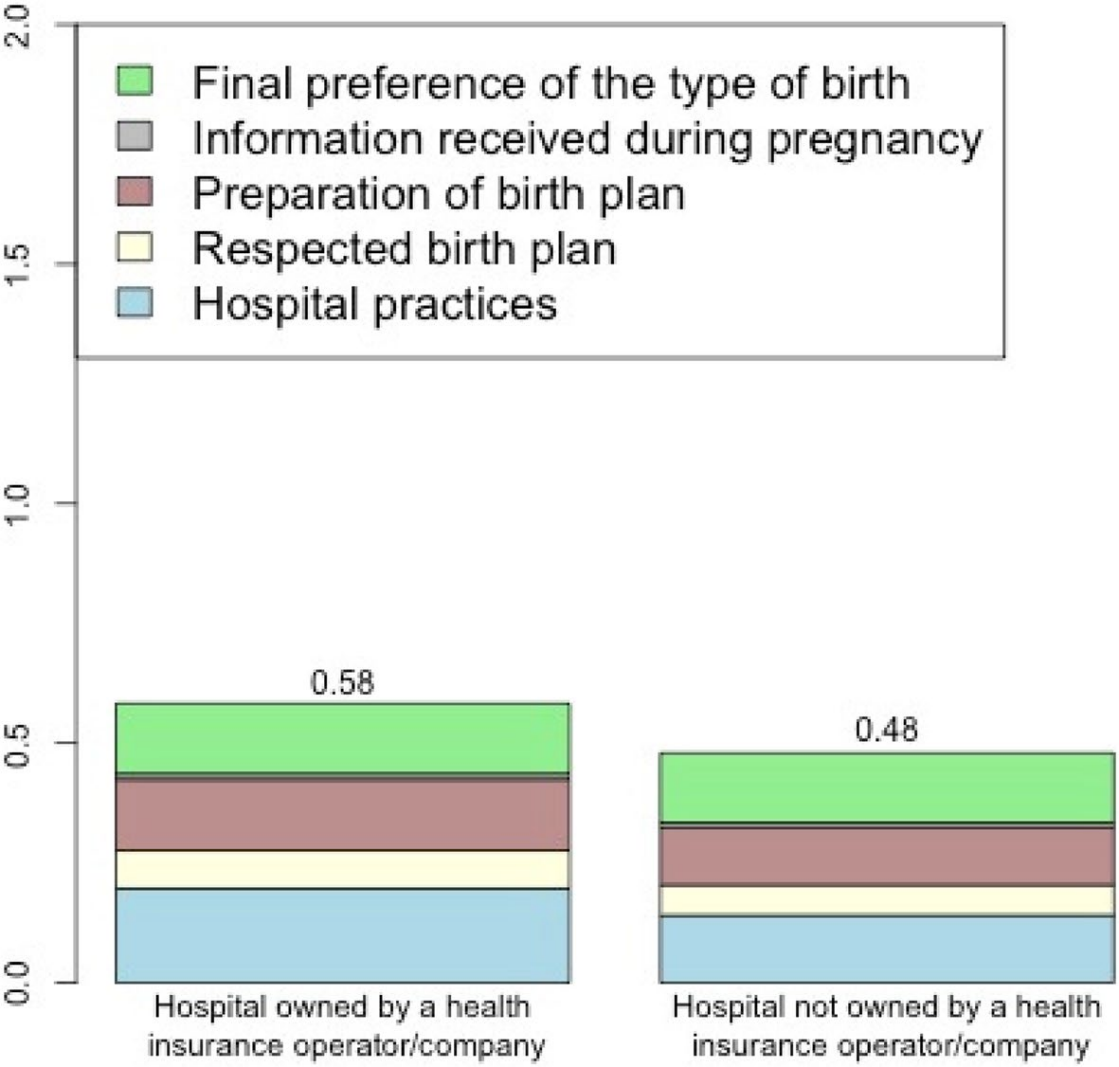
Combined score of “Reorganization of care” and “Participation of women”, according to the type of hospital. Please see Table 1 for more detailed definition of variables

In Fig. 5, the combined score of both components is displayed according to the type of hospital. Hospitals owned by a health insurance company presented the larger scores for both components.

Table 3 shows the predictive probability of vaginal birth among women “Exposed to the PPA model of care” and “Standard of care model” in the 12 hospitals. Hospitals 5 (27%), and 9 (28%) recorded the highest probability of vaginal birth among all women. However, the largest differences when comparing those “Exposed to the PPA model of care” and the “Standard of care model” groups were observed in hospitals 3, 5, 7, and 9 (0.18, 0.17, 0.17 and 0.19, respectively).

**Table 3:** Predictive probability of vaginal birth according to the model of care in the 12 hospitals

Hospital	Global probability*	Exposed to the PPA model of care (n)	Standard of care model (n)	Difference (Exposed to PPA—Standard of care model)
1	0,21	0,31 (74)	0,18 (223)	0,13
2	0,18	0,31 (40)	0,16 (326)	0,15
3	0,21	0,34 (127)	0,16 (246)	0,18
4	0,23	0,29 (218)	0,16 (160)	0,13
5	0,27	0,36 (177)	0,19 (208)	0,17
6	0,24	0,28 (222)	0,16 (138)	0,12
7	0,25	0,33 (172)	0,16 (174)	0,17
8	0,25	0,30 (211)	0,18 (148)	0,12
9	0,28	0,34 (278)	0,15 (105)	0,19
10	0,25	0,33 (173)	0,19 (196)	0,14
11	0,24	0,30 (200)	0,16 (141)	0,14
12	0,26	0,32 (213)	0,17 (152)	0,15
Total	0,24	0,32 (2105)	0,17 (2217)	0,15

*Estimated by the Birth Network

**Fig. 6:**
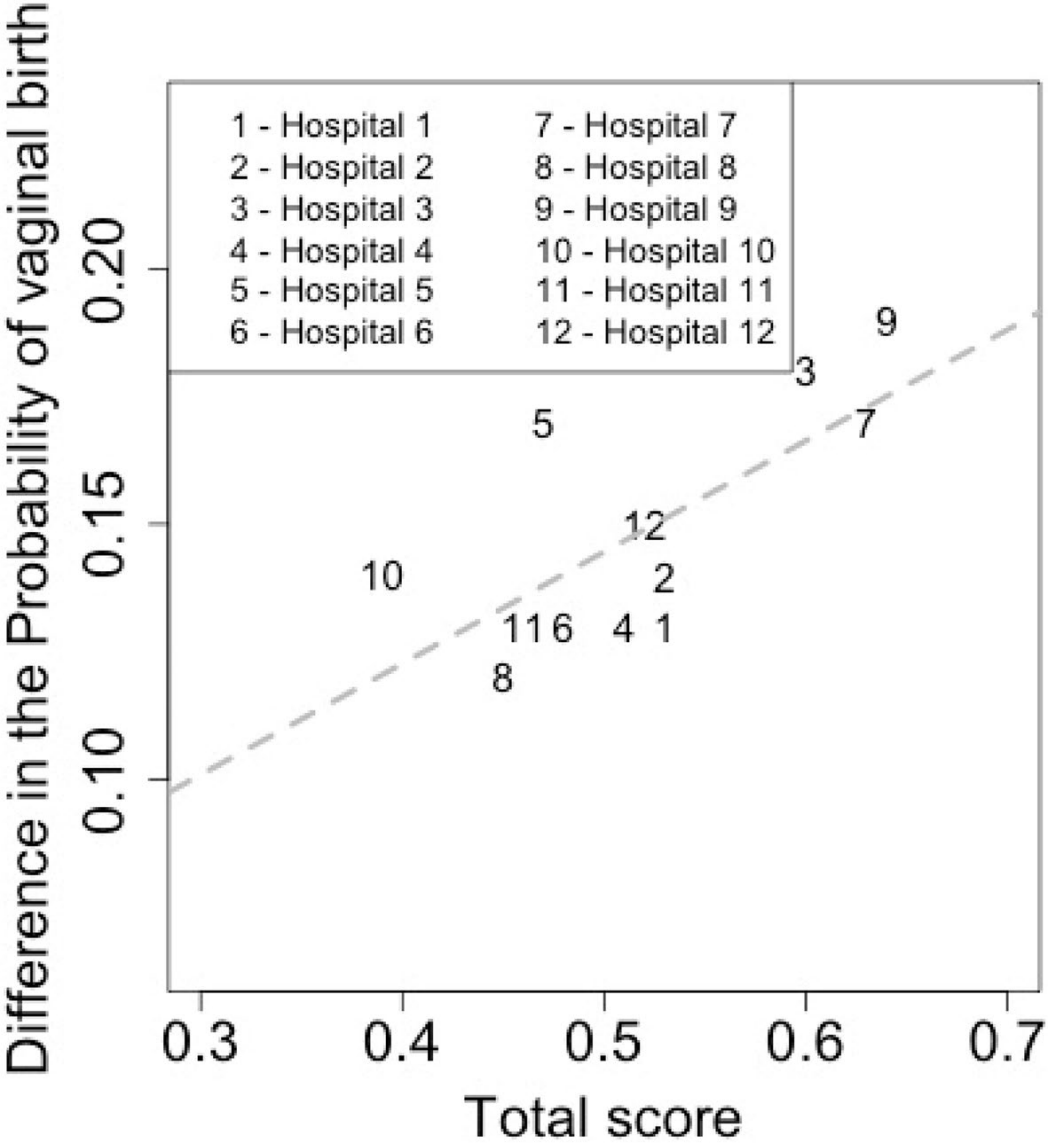
Difference in the probability of vaginal birth according to implementation score

Figure 6 shows the correlation between the global degree of implementation of the two components—“Reorganization of care” and “Participation of women” and the probability of vaginal birth among women “Exposed to the PPA model of care” and those assisted in the “Standard of care model”. Hospitals with higher implementation scores were those with higher differences in the vaginal birth probabilities between groups (correlation of 0.71).

## Discussion

In the twelve hospitals analyzed, PPA had a low implementation score of practices within the “Reorganization of care” and “Participation of women” components. We observed a positive covariation between the implementation of “Reorganization of care” and “Participation of women”. Hospitals owned by a health insurance company presented larger scores for both components. Finally, and most importantly, the probability of vaginal birth was higher in hospitals with a higher degree of implementation of the above components.

The evaluation of multifactorial quality improvement interventions is challenging because of the different contexts and implementation settings. Commonly, changes are decided, yet poorly implemented or not implemented at all [26]. Proposing changes in professional practices and routines in structured environments, such as a private hospital, is not easy. The assessment of the degree of implementation of PPA is an important step to avoiding drawing erroneous conclusions about its effectiveness [27].

PPA is a complex quality improvement project that requires changes in many aspects of the standard model of care in Brazilian private hospitals [16]. This could explain the low degree of implementation of the planned activities, especially for the “Reorganization of care” component, which requires changes in organizational cultures and professional practices. Moreover, PPA demanded that all actors were involved and engaged with the change.

The degree of implementation of PPA activities varied between the 12 hospitals. One reason for this heterogeneity was the different definition of the PPA target population in each hospital, which led to the inclusion of women with different characteristics, thereby affecting the implementation of specific components. For example, primiparous and multiparous women may adhere differently to the project. As would be the case for women of different ages, years of schooling, or those with previous CS and/or chronic conditions. In addition to demographic and obstetric differences, cultural differences in the Brazilian macro-regions regarding childbirth may also affect adherence to the changes proposed by PPA. From phase 2 onwards all hospitals adopted Robson groups 1 to 4 as the target of the PPA. This definition increases homogeneity, but leaves behind group 5, which accounts for a third of cesarean sections in Brazilian private hospitals [28].

The proportion of women targeted by PPA varied among hospitals, which limited its effectiveness. An example was “hospital 2”, where the PPA model of care was applied to only 40 women (10% of women interviewed in this hospital). Although the hospital had the fourth-highest score in the “Reorganization of care” component, the change in vaginal birth rates was minimal (0.18—the worst combined probability of vaginal birth).

The observed positive covariation between the implementation of “Reorganization of care” and “Participation of women” suggests that the implementation of one component could help to improve the other. Hospitals that changed their model of care, encouraging labor and vaginal birth and the use of best practices, were more able to involve women in their own care, as is recommended by recent research [29, 30].

The low implementation of “Participation of women” has two possible explanations. One is related to the difficulties in publicizing the PPA. Only a third of women reported that they knew about the PPA and only 14% of women chose a PPA hospital to give birth at. Other planned activities, such as visiting the hospital before hospital admission for childbirth, participating in antenatal groups, and preparing a birth plan, were infrequent. Another possible explanation is the non-recognition of women as protagonists of their birth process. Studies in the USA have found that mistreatment during childbirth care was exacerbated by unexpected obstetric intervention and by patient-provider disagreement [31], and that women who declined procedures for themselves or their infant reported “poor treatment”, based on a behavior that may be perceived as uncooperative [32]. In Kenya, studies suggest high discordance between women and providers' perspectives in regard to person-centered care experiences, with health care providers recognizing the importance of various aspects of communication and women's autonomy, but failing to provide it for various reasons [33, 34]. Physical, emotional and social support for women can enhance women´s belief in their ability to birth and healthcare professionals need to take cognizance of the empowering effects of the psychological experience of physiological childbirth [35].

Changing the model of care and empowering women and families is difficult in both the public and private sector. However, a study conducted in Brazilian hospitals showed that the private sector was more resistant to the use of evidence-based practices than the public sector [36]. The association between private financing of healthcare and a higher prevalence of CS is not fully understood. Some studies have reported that the main non-clinical factors associated with a high prevalence of CS are the type of financing and/or organization of medical care [37–40], higher education level of women [40], prenatal and childbirth care provided by the same physician [41], characteristics and ambiance of the hospital [42–44], day of the week and time of birth; and low participation of nurses/midwives in childbirth care [45–48]. In Brazil, the private sector has specific organizational characteristics that may favor excess CS which includes: a) the medicalization of childbirth and the perception of C-section as a status symbol b) the “maternity-hotel” model, in which occupancy rates and hospitality are most valued than evidence-based clinical practice; c) the birth as a medical event and obstetrics as an autonomy practice and e) a poorly regulated private healthcare sector. All of these characteristics favor obstetrician convenience [16].

The degree of implementation was higher in hospitals belonging to a health insurance company. This finding was expected, considering the financial interest of healthcare operators to increase vaginal birth rates, which are less costly. In economic analyses conducted in private hospitals in Brazil [49–51], vaginal births were more cost-effective than CS in low-risk pregnancies both in primiparous and in multiparous women without a previous CS.

The probability of vaginal birth in women “Exposed to the PPA model of care” was higher than in those in the “Standard of care model”. This probability increased with rising implementation scores. Previous studies have demonstrated an increase in the use of best practices during childbirth and in the proportion of vaginal births in private hospitals after the implementation of PPA [19, 52]. Borem et al. demonstrated that vaginal delivery increased from 21.5% in 2014 to 34.8% in 2016, a relative increase of 1.62 (95% CI 1.27–2.07, *p* < 0.001), considering 28 hospitals at the end of the first phase of PPA [19]. These results could not be attributed to an overall change in the private sector, as an increase in the proportion of vaginal births was not observed in other private hospitals that did not participate in PPA. All PPA activities are based on non-clinical interventions to reduce cesarean sections and are in line with recent publications that highlight the importance of multi-component and locally-tailored interventions, addressing women (e.g., via birth preparation classes), health professionals (e.g., via the implementation of clinical practice guidelines), and health system and financial factors (e.g., via different payment systems for caesarean section) [30, 53, 54].

This study has some limitations. We used a convenience sample to capture a variety of contextual characteristics with greater explanatory power. However, this sample is not representative of the set of hospitals participating in the PPA. We excluded women with incomplete data from our analysis, which resulted in the loss of 10% of participants. However, most likely, these data are missing completely at random and we do not suspect bias. We also excluded women with hearing and speaking impairments, who are marginalised groups in maternal care and who need to be considered in future studies.

Changes to the model of care in hospitals, such as adjustments to the physical structure, the involvement of nurses/midwives and implementation of clinical guidelines, may have affected women that were not targeted by the PPA. This could have reduced the difference between the compared groups, thereby masking the effect of the PPA.

Finally, we were not able to evaluate the “Monitoring” and “Governance” components of PPA in this quantitative approach. Future evaluations using qualitative data will help build an understanding of the implementation of these components and how they interact with those assessed in this study.

## Conclusion

The degree of implementation of this quality improvement project to increase vaginal births rates was low. This result reflects the difficulties in implementing changes in private hospitals in Brazil. The use of the Bayesian method helped to identify higher scores of implementation in hospitals owned by health insurance companies when compared to other private hospitals, and higher vaginal birth rates in hospitals with higher implementation scores. The PPA is an ongoing quality improvement project and the results demonstrate the need for improvements, especially greater involvement of women and their families.

## Data Availability

The datasets used and/or analyzed during the current study are available from the corresponding author on reasonable request.

## Abbreviations

ANS: National Agency for Supplementary Health
BN: Bayesian Network
CS: Cesarean section
HIAE: Hospital Israelita Albert Einstein
IHI: Institute for Healthcare Improvement
PPA: “Projeto Parto Adequado”
SINASC: Brazilian system for live births
